# Perceived Stigma and Self-Efficacy of Patients With Inflammatory Bowel Disease-Related Stoma in China: A Cross-Sectional Study

**DOI:** 10.3389/fmed.2022.813367

**Published:** 2022-02-16

**Authors:** Yuting Wang, Shuyan Li, Jianfeng Gong, Lei Cao, Dingting Xu, Qiao Yu, Xiaoying Wang, Yan Chen

**Affiliations:** ^1^Center for Inflammatory Bowel Diseases, Department of Gastroenterology, The Second Affiliated Hospital, Zhejiang University School of Medicine, Hangzhou, China; ^2^Center for Inflammatory Bowel Diseases, Department of General Surgery, Jinling Hospital: East Region Military Command General Hospital, Medical School of Nanjing University, Nanjing, China

**Keywords:** inflammatory bowel disease, stoma, self-efficacy, perceived stigma, online peer support group

## Abstract

**Background:**

Patients with inflammatory bowel disease (IBD)-related stoma face physical, psychological, and social adjustment challenges. However, knowledge about stigmatization and self-management, which is important for clinical care and patient education strategies, is lacking.

**Objective:**

To evaluate the level of stigma and self-management ability of Chinese patients with IBD-related stoma using an online questionnaire.

**Methods:**

Participants were recruited from two general hospitals in mainland China and the internet platforms of the China Crohn's and Colitis Foundation (CCCF). Participants completed a questionnaire, which gathered data on sociodemographic, clinical, and experience in online groups, self-efficacy scale, and social impact scale. The influencing factors of self-efficacy and perceived stigma were assessed by stepwise multivariate regression analyses.

**Results:**

One hundred and seventy-six respondents were included. Most of the respondents (78/176, 44.32%) spent between 500 and 999 RMB ($77–153) on ostomy care accessories monthly. Three patients reported using homemade ostomy products. The average score on the self-efficacy scale was 75.79 ± 23.91, which reflected a moderate level of self-efficacy, and 69 (39.2%) respondents had low-level self-efficacy. The average social impact scale score was 62.76 ± 12.69, which reflected a moderate level of perceived stigma. Forty-three (24.43%) patients experienced severe levels of perceived stigma. Stepwise multivariate regression analysis revealed that self-efficacy was associated with educational level (*P* = 0.007), whereas stigma was associated with nursing privacy (*P* = 0.021) and acceptance by the closest person (*P* = 0.005). A total of 91% of respondents who participated in online peer support groups believed the groups were helpful for disease management.

**Conclusions:**

Chinese patients with IBD-related stoma reported a moderate degree of perceived stigma; their level of self-efficacy was low to moderate. High educational level was associated with high self-efficacy. Notably, acceptance of the stoma by the closest person was an influencing factor protecting patients from perceived stigma. Interventions aimed at improving patient education, reducing economic burden, and strengthening social support should be considered to help improve the living conditions of patients with IBD-related stoma.

## Introduction

Inflammatory bowel disease (IBD) is a group of diseases characterized by chronic inflammation of the digestive tract. The prevalence of IBD is increasing globally, and it is expected to continue to increase in the future, with important implications for health and the economy (1). IBD incidence in China, which includes up to 11.6 ulcerative colitis (UC) cases per 100,000 person-years and 1.4 Crohn's disease (CD) cases per 100,000 person-years, is estimated to be the highest in Asia (2–4). Ostomy is an effective and commonly used treatment option for refractory or severe IBD (5). However, post-ostomy changes and complications, such as altered body image, stool leakage, social isolation, ostomy-related dermatitis, sexual dysfunction, psychological distress, and perceived loss of control, result in decreased quality of life (6–8). Given these circumstances, patients with stoma are at risk of experiencing perceived stigma, which describes the degree to which individuals perceive discrimination directed at them or others (9). Perceived stigma is associated with decreases in self-efficacy and is a significant predictor of poorer outcomes in patients with IBD (10). According to a survey, stoma was the most embarrassing complication perceived by patients with IBD (9). A Chinese study reported 44% of participants with colorectal cancer (CRC)-related stoma experienced high levels of stigma (11). However, the level of stigma in patients with IBD-related stoma has not been accurately measured yet.

Research on chronic diseases shows that self-efficacy is an important core concept of self-management and an important predictor of stoma health care management ability (12). Improving the self-management ability of patients with stoma after ostomy is the key to their smooth recovery and adaptation to new life from the perspective of psychosocial factors. Self-management focuses on patient's ability to manage their condition rather than treatment based within the healthcare system or centered on a healthcare professional. Therefore, this management strategy seeks to restore patient's autonomy, position patients at the center of their own management process, and help them acquire and maintain competencies to enable them to efficiently manage their condition (13). From the limited data available, the self-efficacy level of patients with IBD-related stoma is unclear, whereas those with CRC-related stoma showed a moderate level (14).

Rapid development in internet technology and digital interventions (accessed via computers and mobile phones) that provide self-management information has been proposed as a promising mode of self-management intervention (13). Studies have estimated that more than 50% of patients with IBD use the internet as a source of information for disease management (15, 16). More people are participating in online peer-led social support groups, which connect people in similar circumstances and transcend geographical limitations (17–19). The function, importance, and future development strategy of online peer support groups need to be explored further.

To date, no studies have been published on stigma and self-management in patients with IBD-related stoma in China. This study seeks to address these research questions: (1) Is the extent of perceived stigma and self-efficacy in patients with IBD-related stoma consistent with those with CRC-related stoma? (2) Which social and disease factors affect perceived stigma and self-efficacy of patients with IBD-related stoma? (3) Is participating in online groups helpful for patients with IBD-related stoma in disease management?

## Methods

### Study Design and Participants

**Figure 1:**
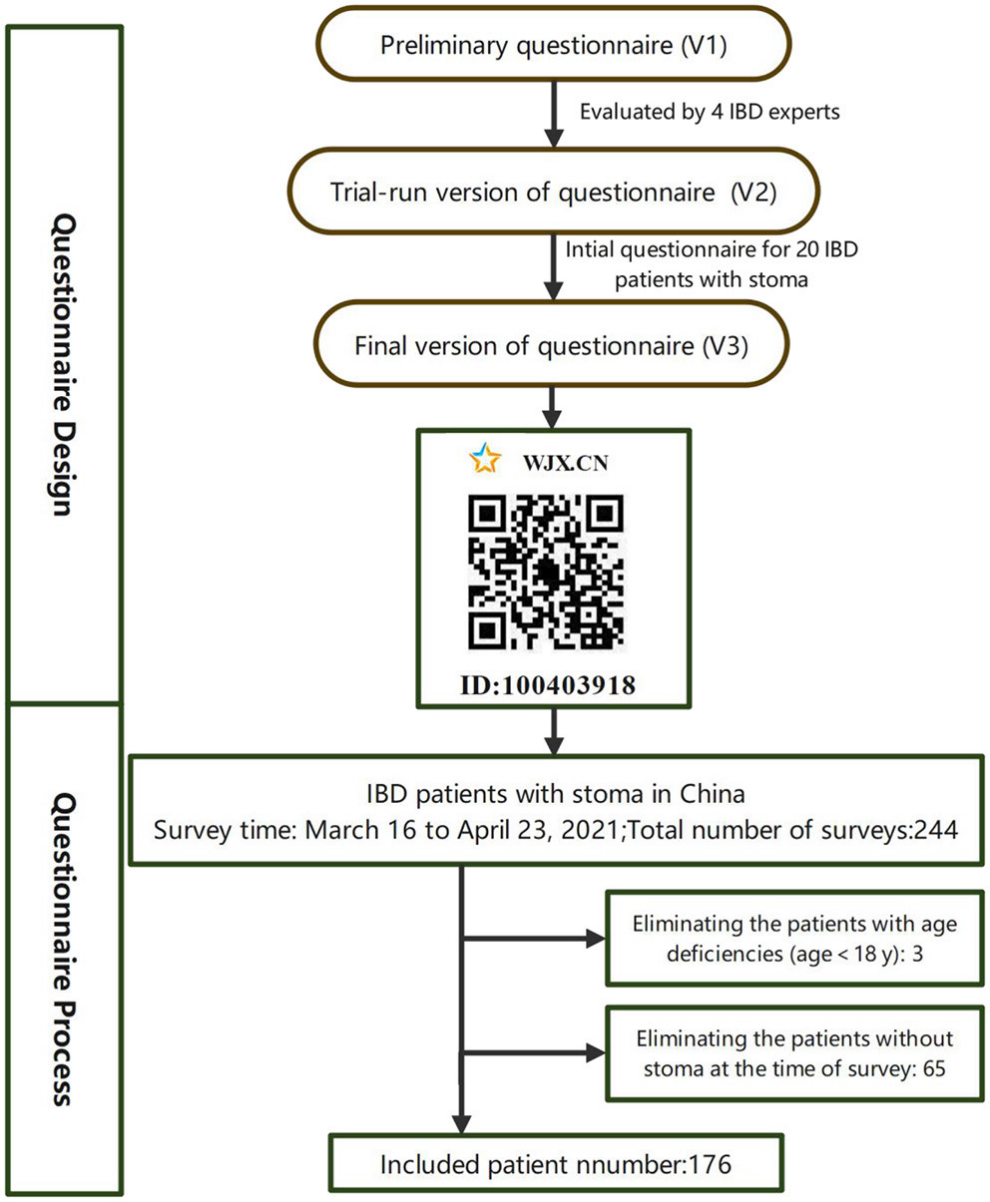
Flow chart depicting the selection of the survey sample for analysis.

A cross-sectional observational study was conducted among Chinese patients with IBD-related stoma through an electronic questionnaire in two hospitals in China from March 16 to April 23, 2021. The questionnaire was produced using Wenjuanxing (20), a professional online questionnaire tool. It was distributed to patients at the IBD clinic of the Second Affiliated Hospital of Zhejiang University Medical College and the general surgery clinic of the General Hospital of Eastern Theater Command. Additionally, the survey was posted on the internet platforms of the China Crohn's and Colitis Foundation (CCCF) to facilitate the collection of questionnaires. The inclusion criteria were patients who: (1) gave informed consent; (2) were 18 years of age or older; (3) diagnosed as CD, UC or indeterminate colitis; (4) with a stoma whether it was a colostomy or an ileostomy and was expected to be permanent or temporary; (5) being able to complete Web-based surveys in Chinese. Diagnoses of IBD were confirmed by reviewing medical records and querying treating physicians directly as needed. Identified charts were reviewed by IBD experts in detail, including clinic notes, hospitalization records, endoscopic evaluations, surgical reports, laboratory testing, microbiological testing, radiologic studies, and pathology results. Patients with questionable IBD diagnoses were excluded. The exclusion criteria included patients with a history of mental illness, cancer or other serious physical illnesses. As shown in Figure 1, the questionnaire was modified based on feedback from the pilot samples and was iteratively refined by IBD experts. This study was approved by the medical ethics committee of the Second Affiliated Hospital of Zhejiang University Medical College (approval number: 2021–0237).

### Measurements

Participants were asked to report the following sociodemographic and clinical information: age, gender, education level, employment status, place of residence, marital status, IBD diagnosis, disease duration, remission status, income, health insurance status. Study data also included type of stoma, time of ostomy, cost of stoma supplies, complications of stoma, body image, privacy of care for the stoma, ostomy leaks and difficulty of stoma care.

Information about social support was obtained, including acceptance of stoma by the closest person, and the main source of psychological support was obtained. Participants were asked to provide information on their experiences in online groups, and if so, they were asked about topics of interest, their activity state, feelings of participation in online groups and whether it's helpful. The subjects who indicated “it's helpful” were further asked about what aspects of online groups were perceived to be helpful. They could choose out of options including “increased sense of belonging,” “gained confidence in the management of stoma or disease,” “helped to made up my mind to undergo ostomies,” “released the negativity and resonated with fellows,” “drew strength from helping others” or fill in any other answers.

The self-efficacy scale by Bekkers (21) in 1996 is currently the most tested and widely used scale for measuring the level of self-efficacy of patients with stoma. The scale comprises 28 items, two dimensions (stoma care self-efficacy and social self-efficacy), and six separate items, which are scored on a 5-point scale (1 = no confidence, 2 = slightly confident, 3 = fairly confident, 4 = highly confident, and 5 = extremely confident). The total score ranges from 28 to 140, and is divided into three grades: ≤ 65 indicates low-level self-efficacy, 66–102 indicates moderate-level self-efficacy, and ≥103 indicates high-level self-efficacy. Wu et al. (22) from Hong Kong developed a Chinese version of the self-efficacy scale for patients with stoma, authorized this study to use the Chinese version. In this study, Cronbach's α coefficient of the scale was 0.926.

The social impact scale (SIS) was developed by Fife and Wright (23) in 2000 and translated into Chinese by Pan et al. (24) in 2007. It was originally used to assess stigma among patients with acquired immune deficiency syndrome (AIDS) and cancer. This scale contains 24 items and examines four domains of perceived stigma—social rejection, financial insecurity, internalized shame, and social isolation. The items are scored from 1 (strongly agree) to 4 (strongly disagree). Average item scores were classified into three levels: mild (1–1.99), moderate (2–2.99), or severe (3–4) stigma. Pan authorized this study to use the Chinese version. In this study, Cronbach's α coefficient was 0.949.

### Statistical Analysis

All statistical analyses were performed using the SPSS software (version 25, IBM Corporation). Demographic data and stoma-related information were summarized as means and standard deviations for continuous variables after testing for normal distribution and as frequency counts (percentages) for categorical variables. The means and standard deviations of stigma and self-efficacy scores were calculated and reported as high, moderate, or low. The mean scores were compared with different characteristics using *t*-tests (for two-level variables, such as gender) or one-way ANOVA (for variables with three or more levels, such as employment status) to determine the associations between demographic data or stoma-related information and dependent variables (self-efficacy or stigma level). Least significant difference corrections were used for *post-hoc* comparisons. An α-level of 0.05 was used to statistical significance.

Multivariate linear regressions (with stepwise variable selection) were used to explore the main factors that influenced stigma or self-efficacy among patients with stoma. Collinearity diagnostics was used to exclude correlations among independent variables. The variables for which the results of univariate linear regression analysis were statistically significant were used as independent variables. The inclusion standard was 0.05, and the removal standard was 0.10.

## Results

### Participant Characteristics

As shown in Figure 1, a total of 244 questionnaires were completed. Only 176 questionnaires were valid. Respondents came from 24 provinces and cities across the country. The male-female ratio of the respondents was almost 1:1(89 males vs. 87 females). The mean age was 37.10 ± 9.98 years (range: 18–64 years). Out of 176 respondents, 156 (87.5%), 16 (9.09%), and 6 (3.41%) respondents reported a diagnosis of CD, UC, and unformed colitis, respectively. Regarding the type of stoma, 97 (55.11%) participants had a temporary stoma and planned to retract it in the future, 70 (39.77%) participants had a permanent stoma, and 9 (5.11%) participants had no idea about the type of stoma. With respect to the surgical procedure participants had undergone, 126 (71.59%), 36 (20.45%), and 5 (2.84%) participants underwent ileostomy, colostomy, and jejunostomy, respectively. Approximately 39.2% of the respondents had been diagnosed with IBD for more than 10 years, whereas, 42.6% had a stoma for 1–5 years.

### Self-Efficacy of Patients With IBD-Related Stoma

**Figure 2:**
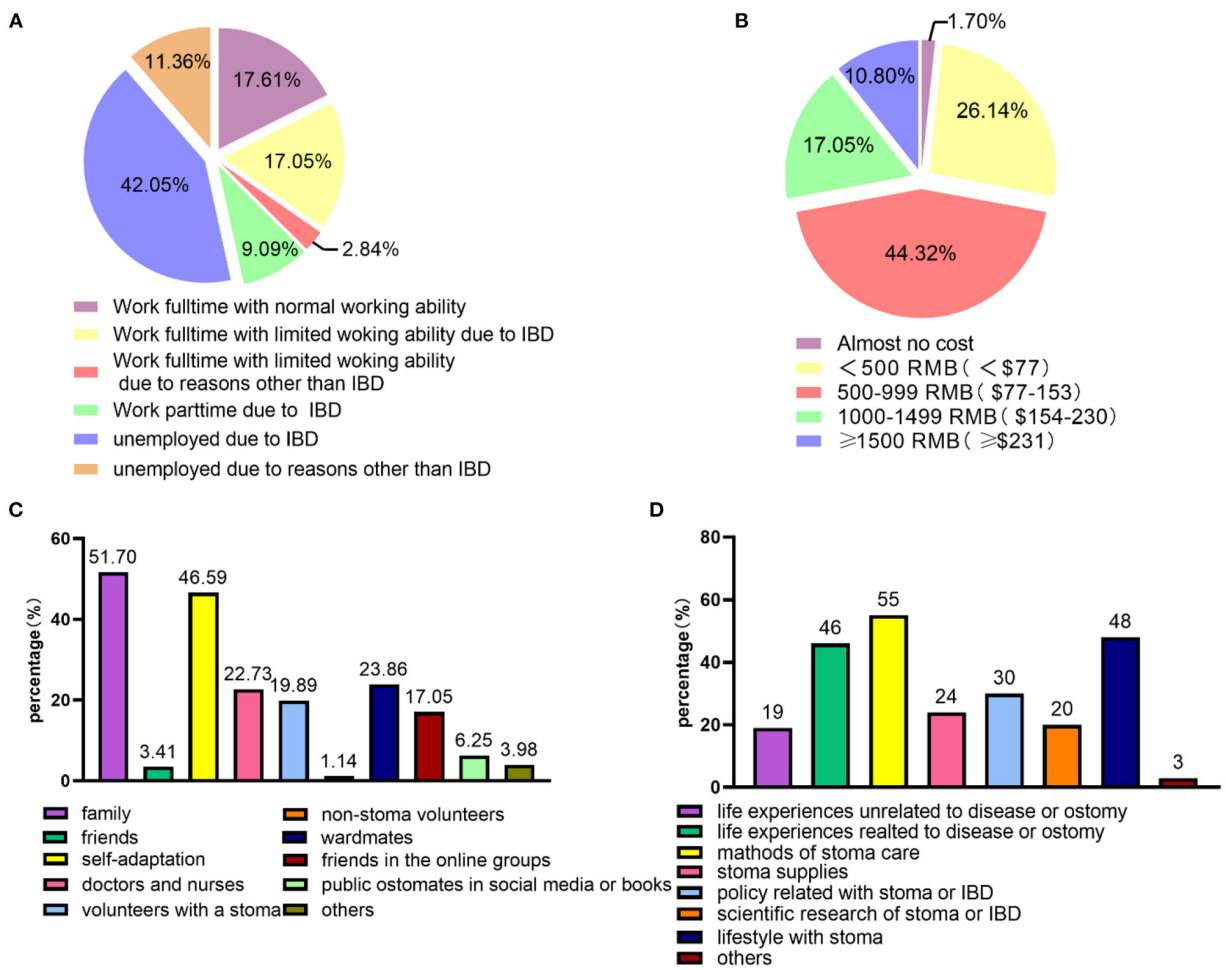
Analysis of the status or feelings of patients with IBD stoma regarding job, costs, physiological support, and online peer support groups. **(A)** Employment status of patients with IBD-related stoma (*n* = 176). **(B)** Costs of ostomy care accessories per month (equivalent to USD, *n* = 176). **(C)** The main source of psychological support after stoma surgery (*n* = 176). **(D)** Topics of interest in online peer support groups (*n* = 100).

Before ostomy, 67 (38.07%) participants decided the location of the stoma with their doctors, whereas 36 (20.45%) participants reported no communication with their doctors. Fifty-nine (33.52%) of participants were only informed of the location of the stoma. The ostomy affected the employment status of participants. As shown in Figure 2A, only 17.61% of participants worked full-time and maintained normal working ability; 9.09% of the participants worked part-time; and 42.05% of the patients were unemployed. Concerning the ostomy care accessories. most of the respondents (78/176, 44.32%) spent between 500 and 999 RMB ($77–153) monthly, whereas 19 (10.8%) of participants spent more than 1,500 RMB ($231). Three patients chose to make homemade ostomy bags (Figure 2B). Additionally, the reimbursement rates for these costs were assessed. Although 34.1% of the respondents indicated that estimation was difficult, the reimbursement rate of most of the remaining patients (73/176, 42.2%) was < 10%. Only 23.69% of the patients exceeded 10%.

The average score of the self-efficacy scale was 75.79 ± 23.91. Sixty-nine (39.2%) respondents had a low level of self-efficacy, 77 (43.75%) had a moderate level of self-efficacy, and 30 (17.05%) had a high level of self-efficacy (17.05%). Table 1 shows the associations between demographic data or stoma-related information and self-efficacy. Significant differences in self-efficacy scores were observed among different groups regarding age, duration of illness, duration of stoma, employment status, monthly household income, education level, membership in online groups, and acceptance of stoma by the closest person.

**Table 1:** Summary of the associations between demographic and stoma-related characteristics and self-efficacy (*n* = 176).

**Characteristic**	***n***	**Self-efficacy Mean ±SD**	***t*/*F*(*P*)^a^**	**Characteristic**	***n***	**Self-efficacy Mean ±SD**	***t/F(P)***
**Gender**				**Place of residence**			
Male	89	77.27 ± 23.64	0.829(0.912)	City	119	76.99 ± 23.28	0.952(0.337)
Female	87	74.27 ± 24.22		Village	57	73.28 ± 25.19	
**Age (years)**				**Employment status**			
18–29	32	84.42 ± 22.81	6.118(*0.003*)	Full-time	66	85.53 ± 24.09	6.268(*0.002*)
30–49	126	76.03 ± 23.85		Part-time	16	75.91 ± 14.16	
≥50	18	59.98 ± 23.76		Unemployed	94	70.33 ± 23.77	
**Duration of IBD (years)**				**Education level**			
<1	11	67.03 ± 20.26	2.843(*0.026*)	Primary school	8	66.98 ± 12.43	7.719(<*0.001*)
1–2.99	25	71.86 ± 21.74		Junior high school	45	61.46 ± 18.82	
3–4.99	21	90.17 ± 28.80		High/technical secondary school	38	77.64 ± 27.44	
5–9.99	50	72.32 ± 21.07		Junior college	39	85.96 ± 22.11	
≥10	69	76.74 ± 24.25		College or beyond	46	81.51 ± 22.39	
**Duration of stoma (years)**				**Participation in activities with other stoma patients**			
<0.25	13	64.67 ± 19.36	3.293(*0.022*)	Yes	17	81.27 ± 26.45	0.995(0.321)
0.25–0.99	42	73.04 ± 19.21		No	159	75.20 ± 23.64	
1–5	75	81.80 ± 26.30		**Joined online groups of stoma patients**			
≥5	46	71.63 ± 21.99		Yes	100	79.53 ± 23.24	2.413(*0.017*)
**Monthly household income (equivalent to US$)**				No	76	70.87 ± 24.03	
<2,000 (< $308)	26	70.47 ± 19.75	5.228(<*0.001*)	**Acceptance of the** **stoma by people closest to you**			
2,000–4,999 ($308–771)	69	69.76 ± 22.27		Not at all accepted	10	59.98 ± 14.65	16.289(<*0.001*)
5,000–9,999 ($772–1,542)	51	77.63 ± 23.29		Not accepted	22	60.07 ± 15.93	
≥10,000 (≥$1,543)	30	91.13 ± 25.57		Basically accepted	97	72.08 ± 21.36	
				Completely accepted	47	94.68 ± 23.85	

a *Values in italics are significant at P < 0.05*.

### Perceived Stigma of Patients With IBD-Related Stoma

Most patients felt that their relatives accepted the stoma (88 accepted basically and 45 accepted completely), whereas 15 (8.52%) participants reported that relatives did not accept well, 7 (3.98%) participants were certain their relatives were unaccepting of the stoma.

The average score of the SIS scale was 62.76 ± 12.69. Twelve (6.82%) patients had mild levels of perceived stigma, 121 (68.75%) patients had moderate levels of perceived stigma, and 43 (24.43%) patients had severe levels of perceived stigma. Table 2 shows the associations between demographic data or stoma-related information and perceived stigma. Significant differences in stigma scores were observed among different groups of patients regarding age, residence in rural areas, monthly household income, privacy of stoma care, image satisfaction, education level, presence of pocket leakage, stoma complications, and acceptance of the stoma by the closest person.

**Table 2:** Summary of the associations between demographic and stoma-related characteristics and perceived stigma (*n* = 176).

**Characteristic**	***n***	**Stigma Mean ±SD**	**t/F(*P*)^**a**^**	**Characteristic**	***n***	**Stigma Mean ±SD**	**t/F(*P*)**
**Type of stoma (classified by time)**				**Place of residence**			
Temporary	97	62.31 ± 12.03	−0.446(0.656)	City	119	61.06 ± 12.18	−2.615(*0.01*)
Permanent	70	63.21 ± 14.11		Village	57	66.32 ± 13.09	
**Age (years)**				**Pocket leakage in past 3 months**			
18–29	32	65.93 ± 14.43	0.957(0.386)	Yes	116	64.50 ± 12.63	2.568(*0.011*)
30–49	126	62.31 ± 11.91		no	60	59.40 ± 12.21	
≥50	18	63.72 ± 13.78					
**Educational level**				**Monthly household income (equivalent to US$)**			
Primary school	8	68.38 ± 12.27	4.399(*0.002*)	<2,000(< $307)	26	69.50 ± 13.47	5.903(<*0.001*)
Junior high school	45	68.58 ± 11.78		2,000–4,999($308–771)	69	65.14 ± 12.33	
High/technical secondary school	38	60.32 ± 13.31		5,000–9,999($772–1,542)	51	59.71 ± 10.61	
Junior college	39	58.79 ± 10.02		≥10,000(≥$1,543)	30	56.63 ± 12.45	
College or beyond	46	61.78 ± 13.38					
**Acceptance of the stoma by people closest to you**				**Privacy of care for the stoma**			
Not at all accepted	10	71.00 ± 7.44	13.751(<*0.001*)	Cannot be protected at all	11	71.86 ± 16.60	8.469(<*0.001*)
Not accepted	22	73.53 ± 16.27		Mostly cannot be protected	33	70.62 ± 11.82	
Mostly accepted	97	63.17 ± 10.43		Mostly can be protected	114	60.47 ± 11.65	
Completely accepted	47	54.47 ± 11.34		Completely can be protected	18	54.46 ± 10.82	
**Body image**				**Have ever had Complications**			
Not at all satisfied	68	68.63 ± 14.02	11.53(<*0.001*)	Yes	108	64.51 ± 10.76	2.33(*0.021*)
Not satisfied	60	62.21 ± 9.88		no	68	59.99 ± 14.92	
Well enough	43	55.95 ± 9.98					
Satisfied	5	48.00 ± 5.00					

a *Values in italics are significant at P < 0.05*.

### Influencing Factors of Perceived Stigma and Self-Efficacy

Multivariate stepwise linear regression analysis showed that age (β = −0.211, *P* < 0.001), stigma (β = −0.555, *P* < 0.001), and educational level (college or above vs. primary school) (β = 3.388, *P* = 0.007) were the main factors affecting the self-efficacy of patients with IBD-related stoma (Table 3). Self-efficacy (β = −0.524, *P* < 0.001), nursing privacy (β = 0.146, *P* < 0.001), acceptance of the stoma by the closest person (β = −0.178, *P* = 0.005), and age (β = −0.132, *P* = 0.029) were the main factors affecting stigma in patients with IBD-related stoma (Table 4).

**Table 3:** Factors influencing self-efficacy among patients with IBD-related stoma.

**Model**	**B**	**β**	***T*-value**	***P*-value^**a**^**
Constant	156.238		14.824	<*0.001*
Stigma	−1.046	−0.555	−9.329	<*0.001*
Educational level (Junior high school and high/technical secondary school)	6.158	0.105	1.558	0.121
Educational level (college or above)	9.310	3.388	2.748	*0.007*
Age	−0.551	−0.211	−3.569	<*0.001*

a *F = 35.254; R^2^ = 0.452; values in italics are significant at P < 0.05*.

**Table 4:** Factors influencing stigma among patients with IBD-related stoma.

**Model**	**B**	**β**	***T*-value**	***P*-value^**a**^**
Constant	93.998		20.228	<*0.001*
Self-efficacy	−0.278	−0.524	−7.955	<*0.001*
Nursing privacy	4.208	0.146	2.324	*0.021*
Acceptance of the stoma by the closest person	−5.567	−0.178	−2.840	*0.005*
Age	−0.183	−0.132	−2.207	*0.029*

a *F = 34.191; R^2^ = 0.444; values in italics are significant at P < 0.05*.

### Social Support and Online Peer Support Groups

“The most important source of psychological support or the main driving force for you to accept the stoma” was a multiple-choice question, and respondents could choose three choices at most. As shown in Figure 2C, the most popular choice was “family” (91/176, 51.7%), followed by “self-adaptation” (82/176, 46.59%). Some patients received psychological support from ward mates (42/176, 23.86%), doctors and nurses (40/176, 22.73%), volunteers with a stoma (35/176, 19.89%), and friends in the online groups (30/176, 17.05%).

In this survey, 100 respondents had experiences with online peer support groups. Further investigation revealed that most of them were “diving” in group discussions, barely interacted with others but paid attention to the events happening in the group (42/100, 42%), or occasionally expressed ideas and opinions on topics of interest (41/100, 41%). Regarding which messages or discussions were of much interest, discussions on the methods of stoma care were the most popular (55/100, 55%), followed by discussions on lifestyle modifications of patients with stoma, such as diet and exercise (48/100, 48%), and discussions on disease or ostomy-related life experiences of other patients (46/100, 46%) (Figure 2D). Of the 100 participants, 91 participants believed that group participation was helpful for individual disease or stoma management. This finding was corroborated by the finding that group participants helped fellow patients increase their sense of belonging (65/91, 71.43%) or their confidence in the management of stoma or disease (63/91, 69.23%). For some patients (19/91, 20.88%), participating in the group helped them decide on undergoing ostomies.

## Discussion

According to previous studies 0.66 and 1.5% of patients with IBD in the United Kingdom and United States undergo ostomies, respectively, but no authoritative statistics on IBD are available in China (25, 26). Patients with stoma represent a minor proportion of patients with IBD in China, according to our clinical practice conditions. Additionally, the current status of such patients are poorly recognized. Psychosocial assessments of patients with stoma have been increasingly performed in recent years, but the study population predominantly included patients with CRC (11, 14). Few studies have been conducted exclusively on patients with IBD-related stoma. To our knowledge, this study is the first to assess the levels of self-efficacy and stigma in patients with IBD-related stoma.

In this study, the stigma level of patients with IBD-related stoma was consistent with that of patients with CRC-related stoma in other studies evaluated with the same SIS scale (11, 27). However, the average self-efficacy score seems to be slightly lower than the measurement level of the same scale for patients with CRC (75.79 ± 23.91 vs. 81.03 ± 16.30) (14). One obvious difference was that patients with IBD-related stoma were younger than those with CRC-related stoma (37.10 ± 9.98 vs. 60.95 ± 7.38) (14). Moreover, patients with IBD-related stoma usually have a longer course before surgery than patients with CRC-related stoma. Additionally, ostomy may only be a clinical remission rather than a cure; therefore, they need to prepare mentally to cope with the disease for life. Therefore, the present situation of patients with IBD-related stoma should be understood.

Our previous study revealed that economic burden is a major difficulty faced by Chinese patients with IBD, and low-income patients have worse disease control (28). Another Chinese study showed that 30.6% of patients with IBD spent over half of their income on medical costs, which is an independent factor that impairs their quality of life (29). Consistent with these results, 96.02% (169/176) of participants thought that a certain degree of economic burden is caused by disease and treatment in this study. Further analysis found higher self-efficacy (*P* < 0.01) and lower stigma scores (*P* < 0.01) among participants with monthly family income more than 10,000 RMB ($1,545) compared with those with family monthly income less than this value. The economic burden to patients with IBD-related stoma is substantial and cannot be ignored. Majority of the participant (108/176, 61.36%) in our survey experienced a stoma complication, which means more additional associated costs, not to mention that re-operation is needed in some cases. Ostomy products are consumables and require regular replacement, but the highest proportion of respondents (73/176, 42.2%) in this study reported the reimbursement ratio for cost is <10%. Three respondents reported using their homemade ostomy products made of polythene bags and rubber rings to save money.

Educational level is one of the key factors that influence self-efficacy. Understanding that patients with higher educational levels are more likely to accept various issues in daily life, obtain pertinent medical knowledge and information, and use their knowledge and skills to solve problems, is not difficult. Economic and educational factors may partly explain why rural residents have a stronger sense of stigma than urban residents in this survey (*P* = 0.01). Compared with urban residents, rural populations have a lower proportion of medical insurance payments, have more restricted access to health care, and are relatively lacking in educational resources (30). Due to IBD (74/176, 42.05%) or other reasons (20/176, 11.36%), more than half of the respondents were unemployed. The self-efficacy score of full-time patients with stoma was higher than that of unemployed patients (*P* < 0.01). On the one hand, employment status directly affects economic resources and the ability to fund one's lifestyle establishes confidence and self-worth, as these patients can undertake social and family responsibilities. On the other hand, employment status affects their participation in community life, affecting their sense of belonging and ability to address challenges.

In this survey, the main source of postoperative psychological support for patients with stoma was family (91/176, 51.7%), which is related to the strong traditional family concept in China. Family members are highly interdependent, take care of each other, and fulfill their family relationship obligations. Social support mainly refers to emotional and material help and assistance from family, relatives, friends, and other members of society, such as colleagues, organizations, groups, and communities, which reflects the closeness and quality of one's social connections (31). Studies have shown that social support has an irreplaceable role in maintaining the stability of life for patient with stoma (31), and the ability to take care of stoma is related to the level of support received and emotional “readiness” (32). Our study confirmed that acceptance of stoma by the closest person remained as a significant factor for reduced stigma in the multivariate regression analysis.

Additionally, peer support is a form of social support, which is provided by specific groups with the same experience or similar demographic characteristics by sharing experiences or transmitting information (33). Online social networking represents a prominent form of communication in many people's lives. For individuals with stigmatized illnesses or difficulties in in-person interpersonal communication, social media makes connection with others who share similar health conditions to seek or disclose health information without revealing one's personal identity possible (34). As in this survey, 42% of the respondents were fully participating in the internet group's activities. Compared with spontaneous in-person encounters, social media users maintain greater control, meaning that they can choose their own level of engagement and the extent to which they interact with others. Studies have confirmed that identifying with a social group can improve self-esteem and self-efficacy and reduce uncertainty (35). Consistent with these findings, the self-efficacy of patients who joined the online peer support group in this survey was higher than that of patients who did not (*P* < 0.05).

Among the respondents who joined the online support group, 91% of participants found the experience helpful to the management of disease or stoma by increasing the sense of belonging (65/91, 71.43%) and enhancing confidence in the stoma management (63/91, 69.23%). This finding is consistent with other results indicating that peer support promotes positive emotional support, allows venting of negative emotions, and helps patients find strategies to deal with challenging environments (36, 37). The fear and worry about ostomy is common among patients with IBD (26), but 20.88% of patients in this study agreed that the online peer support group gave them the courage and determination to undergo surgery. Among the online group discussion topics, the most popular were stoma nursing methods (55/100, 55%), lifestyle (48/100, 48%), and peer experience (46/100, 46%), which suggested that teaching and resources from medical staff are practical for stoma management, but they may be far inferior to peer patients in emotional value.

Stoma care is not a simple task, as patients need to adjust their diet, clothing, exercise, and other daily life activities. Additionally, they need to learn to change stoma pouches, handle excrement, prevent complications, and adapt their social lives. Few patients with IBD have stoma; therefore, regarding enterostomy, patients with CRC are more considered than patients with IBD. This is the first nationwide survey of patients with IBD-related stoma to assess patient's level of stigma and self-efficacy in China. Our findings reflect the current situation of Chinese patients with IBD stoma, and specialist physicians and nurses need to understand their specific situations. The results of this study are helpful in identifying patients prone to low self-efficacy or severe stigma in the clinic for timely adjustment of medical measures and provide individualized care. In addition, this study investigated the participation and interaction characteristics of patients with IBD stoma in online peer support groups to deepen our understanding of the role of online peer support. The study findings may be significant to the future planning of CCCF online group projects.

This study had few limitations. First, the cross-sectional study design precluded the possibility of ascertaining causality between risk factors and self-efficacy or stigma. Second, few patients with IBD have stoma; therefore, the study could not gather a large sample size. Third, the questionnaire was completed online; therefore, patients with limited access to the internet could not be included in our study, and these patients were likely to have poor economic power and disease control. Therefore, selection bias could be present in this study.

## Conclusions

Chinese patients with IBD-related stoma reported a moderate sense of stigma and a low-to-moderate level of self-efficacy. Patients with stoma who completed higher education (college and above) had higher levels of self-efficacy. Additionally, this survey revealed that financial burden may have a direct effect on quality of stoma care. Therefore, strengthening patient education and reducing economic burden may be the keys to improving this situation. Patients whose relatives were not accepting of their condition experienced high levels of stigma. Online peer support groups are worthy of further promotion, as social support may have a significant impact on stigma among patients with IBD-related stoma.

## Data Availability Statement

The original contributions presented in the study are included in the article/supplementary material, further inquiries can be directed to the corresponding author/s.

## Ethics Statement

The studies involving human participants were reviewed and approved by the Medical Ethics Committee of the Second Affiliated Hospital of Zhejiang University Medical College. The patients/participants provided their written informed consent to participate in this study.

## Author Contributions

YC and YW conceived and designed the project. SL, JG, LC, and XW collected the data. YW and QY analyzed the data. YW, DX, and YC wrote the manuscript. All authors read and approved the final manuscript.

## Conflict of Interest

The authors declare that the research was conducted in the absence of any commercial or financial relationships that could be construed as a potential conflict of interest.

## Publisher's Note

All claims expressed in this article are solely those of the authors and do not necessarily represent those of their affiliated organizations, or those of the publisher, the editors and the reviewers. Any product that may be evaluated in this article, or claim that may be made by its manufacturer, is not guaranteed or endorsed by the publisher.

## Abbreviations

AIDS: Acquired immune deficiency syndrome
CCCF: China Crohn's & Colitis Foundation
CD: Crohn's disease
CRC: Colorectal cancer
IBD: Inflammatory bowel disease
SIS: Social impact scale
UC: Ulcerative colitis.

## References

[1] Alatab S Sepanlou SG Ikuta K. The global, regional, and national burden of inflammatory bowel disease in 195 countries and territories, 1990–2017: a systematic analysis for the Global Burden of Disease Study 2017. Lancet Gastroenterol Hepatol. (2020) 5:17–30. 10.1016/S2468-1253(19)30333-4 31648971 PMC7026709

[2] Population density and risk of inflammatory bowel disease: a prospective population-based study in 13 countries or regions in asia-pacific. Am J Gastroenterol. (2018) 114:107–15. 10.1038/s41395-018-0233-2 30177785

[3] Kaplan GG Ng SC. Understanding and preventing the global increase of inflammatory bowel disease. Gastroenterology. (2017) 152:313–21. 10.1053/j.gastro.2016.10.020 27793607

[4] Ng SC Zeng Z Niewiadomski O Tang W Bell S Kamm MA. Early course of inflammatory bowel disease in a population-based inception cohort study from 8 countries in Asia and Australia. Gastroenterology. (2016)150:86–95. 10.1053/j.gastro.2015.09.005 26385074

[5] Tom Ø Bemelman WA Sampietro GM Spinelli A Windsor A Ferrante M. European evidence based consensus on surgery for ulcerative colitis. J Crohns Colitis. (2015) 9:4–25. 10.1016/j.crohns.2014.08.012 25304060

[6] Ross L Abild-Nielsen AG Thomsen BL Karlsen RV Boesen EH Johansen C. Quality of life of Danish colorectal cancer patients with and without a stoma. Support Care Cancer. (2007) 15:505–13. 10.1007/s00520-006-0177-8 17103196

[7] Liao C Qin Y. Factors associated with stoma quality of life among stoma patients. Int J Nurs Sci. (2014) 1:196–201. 10.1016/j.ijnss.2014.05.007

[8] Sarabi N Navipour H Mohammadi E. Relative tranquility in ostomy patients' social life: a qualitative content analysis. World J Surg. (2017) 41:2136–42. 10.1007/s00268-017-3983-x 28321552

[9] Guo L Rohde J Farraye FA. Stigma and disclosure in patients with inflammatory bowel disease. Inflamm Bowel Dis. (2020) 26:1010–6. 10.1093/ibd/izz260 32556190

[10] Taft TH Keefer L Leonhard C Nealon-Woods M. Impact of perceived stigma on inflammatory bowel disease patient outcomes. Inflamm Bowel Dis. (2009) 15:1224–32. 10.1002/ibd.20864 19180581 PMC2938734

[11] Yuan JM Zhang JE Zheng MC Bu XQ. Stigma and its influencing factors among Chinese patients with stoma. Psychooncology. (2018) 27:1565–71. 10.1002/pon.4695 29508500

[12] Seo HW. Effects of the frequency of ostomy management reinforcement education on self-care knowledge, self-efficacy, and ability of stoma appliance change among Korean hospitalised ostomates. Int Wound J. (2019) 16:21–8. 10.1111/iwj.13047 30793857 PMC7948817

[13] Nicholl BI Sandal LF Stochkendahl MJ McCallum M Suresh N Vasseljen O. Digital support interventions for the self-management of low back pain: a systematic review. J Med Internet Res. (2017) 19:e179. 10.2196/jmir.7290 28550009 PMC5466697

[14] Jin Y Ma H Jimenez-Herrera M. Self-disgust and stigma both mediate the relationship between stoma acceptance and stoma care self-efficacy. J Adv Nurs. (2020) 76:2547–58. 10.1111/jan.14457 32700799

[15] Cima R Anderson KJ Larson DW Dozois EJ Hassan I Sandborn WJ. Internet use by patients in an inflammatory bowel disease specialty clinic. Inflamm Bowel Dis. (2007) 13:1266–70. 10.1002/ibd.20198 17567877

[16] Internet use among inflammatory bowel disease patients: an Italian multicenter survey. Eur J Gastroenterol Hepatol. (2009) 21:1036–41. 10.1097/MEG.0b013e328321b112 19543105

[17] Zhaohua D Ziying H Ren C Zhang W Xiang F. What predicts patients' adoption intention toward mhealth services in China: empirical study. JMIR mHealth uHealth. (2018) 6:e172. 10.2196/mhealth.9316 30158101 PMC6135967

[18] Greene JA Choudhry NK Kilabuk E Shrank WH. Online social networking by patients with diabetes: a qualitative evaluation of communication with facebook. J Gen Intern Med. (2011) 26:287–92. 10.1007/s11606-010-1526-3 20945113 PMC3043192

[19] Smith-Merry J Goggin G Campbell A McKenzie K Ridout B Baylosis C. Social connection and online engagement: insights from interviews with users of a mental health online forum. JMIR Ment Health. (2019) 6:e11084. 10.2196/11084 30912760 PMC6454344

[20] *Wenjuanxing: Platform For Survey Design*. Available online at: https://www.wjx.cn.

[21] Bekkers M Knippenberg FV Van D Berge-Henegouwen GV. Prospective evaluation of psychosocial adaptation to stoma surgery: the role of self-efficacy. Psychosom Med. (1996) 58:183–91. 10.1097/00006842-199603000-00013 8849636

[22] Wu KM Chau PC Twinn S. Self-efficacy and quality of life among stoma patients in Hong Kong. Cancer Nurs. (2007) 30:186–93. 10.1097/01.NCC.0000270704.34296.86 17510581

[23] Wright F. The dimensionality of stigma: a comparison of its impact on the self of persons with HIV/AIDS and cancer. J Health Soc Behav. (2000) 41:50–67. 10.2307/2676360 10750322

[24] Ay-Woan Pan LC Betsy L. Fife and ping-chuan hsiung, evaluation of the psychometrics of the social impact scale:a measure of stigmatization. Int J Rehabil Res. (2007) 30:235–8. 10.1097/MRR.0b013e32829fb3db 17762770

[25] Mowat C Cole A Windsor A Ahmad T Arnott I Driscoll R. Guidelines for the management of inflammatory bowel disease in adults. Gut. (2011) 60:571–607. 10.1136/gut.2010.224154 21464096

[26] Beddy D Dozois EJ Pemberton JH. Perioperative complications in inflammatory bowel disease. Inflamm Bowel Dis. (2011) 17:1610–9. 10.1002/ibd.21504 21674718

[27] Qin F Zhen L Ye X Wei H Zhu M Chen J. Stigma and its influence on patients with temporary ostomy: a cross-sectional survey. J Wound Ostomy Continence Nurs. (2020) 47:244–8. 10.1097/WON.0000000000000645 32384528

[28] Yu Q Zhu C Feng S Xu L Hu S Chen H. Economic burden and health care access for patients with inflammatory bowel diseases in China: web-based survey study. J Med Internet Res. (2021) 23:e20629. 10.2196/20629 33399540 PMC7815453

[29] Luo XP Mao R Chen BL. Over-reaching beyond disease activity: the influence of anxiety and medical economic burden on health-related quality of life in patients with inflammatory bowel disease. Patient Prefer Adherence. (2017) 11:23–31. 10.2147/PPA.S118589 28053510 PMC5189695

[30] Meng Q Fang H Liu X Yuan B Xu J. Consolidating the social health insurance schemes in China: towards an equitable and efficient health system. Lancet. (2015) 386:1484–92. 10.1016/S0140-6736(15)00342-6 26466052

[31] Ito N Kazuma K. Factors associated with the feeling of stability in the daily life among colostomy patients. Japan J Nurs Sci. (2010) 2:25–31. 10.1111/j.1742-7924.2005.00029.x

[32] Thorpe G Arthur A McArthur M. Adjusting to bodily change following stoma formation: a phenomenological study. Disabil Rehabil. (2016) 38:1791–802. 10.3109/09638288.2015.1107768 26930444

[33] Kornhaber R Wilson A Abu-Qamar M McLean L Vandervord J. Inpatient peer support for adult burn survivors-a valuable resource: a phenomenological analysis of the Australian experience. Burns. (2014) 41:110–7. 10.1016/j.burns.2014.05.003 24927991

[34] Berger M Wagner TH Baker LC. Internet use and stigmatized illness. Soc Sci Med. (2005) 61:1821–7. 10.1016/j.socscimed.2005.03.025 16029778

[35] Mckenna K Bargh JA. Coming out in the age of the Internet: Identity \“demarginalization\” through virtual group participation. J Personal Soc Psychol. (1998) 75:681–94. 10.1037/0022-3514.75.3.681

[36] Lauritzen J Pedersen PU Bjerrum MB. The meaningfulness of participating in support groups for informal caregivers of older adults with dementia: a systematic review. JBI Database System Rev Implement Rep. (2015) 13:373–433. 10.11124/01938924-201513060-00018 26455756

[37] Vitaliano P Katon W Unützer J. Making the case for caregiver research in geriatric psychiatry. Am J Geriatr Psychiatry. (2005) 13:834–43. 10.1097/00019442-200510000-00002 16223961

